# SYNERGIC TRIAL (SYNchronizing Exercises, Remedies in Gait and Cognition) a multi-Centre randomized controlled double blind trial to improve gait and cognition in mild cognitive impairment

**DOI:** 10.1186/s12877-018-0782-7

**Published:** 2018-04-16

**Authors:** Manuel Montero-Odasso, Quincy J. Almeida, Amer M. Burhan, Richard Camicioli, Julien Doyon, Sarah Fraser, Karen Li, Teresa Liu-Ambrose, Laura Middleton, Susan Muir-Hunter, William McIlroy, José A. Morais, Frederico Pieruccini-Faria, Kevin Shoemaker, Mark Speechley, Akshya Vasudev, G. Y. Zou, Nicolas Berryman, Maxime Lussier, Leanne Vanderhaeghe, Louis Bherer

**Affiliations:** 1 Department of Medicine, Division of Geriatric Medicine, Schulich School of Medicine & Dentistry, University of Western Ontario, London, Canada; 2 Department of Epidemiology and Biostatistics, Schulich School of Medicine & Dentistry, University of Western Ontario, London, Canada; 3 Gait and Brain Lab, Parkwood Institute, Lawson Health Research Institute, London, Canada; 4 Sun Life Financial Movement Disorders Research Centre, Wilfrid Laurier University, Waterloo, Canada; 5 Schulich School of Medicine and Dentistry, University of Western Ontario, London, Canada; 6 Geriatric and Cognitive Neurology, University of Alberta, Edmonton, Canada; 7 Functional Neuroimaging Unit, University of Montreal, Montreal, Canada; 8 Department of Psychology-University of Ottawa, Ottawa, Canada; 9 Department of Psychology and PERFORM Centre, Concordia University, Montreal, Canada; 10 Department of Physical Therapy, University of British Columbia, Centre for Hip Health and Mobility, and Djavad Mowafaghian Centre for Brain Health, Vancouver Coastal Research Institute, University of British Columbia, Vancouver, Canada; 11 Department of Kinesiology, University of Waterloo, Waterloo, Canada; 12 School of Physical Therapy, University of Western Ontario, London, Canada; 13 Division of Neurology and Department of Medicine, University of Toronto. Tanz Centre for Research in Neurodegenerative Diseases, University of Toronto, Toronto, Canada; 14 Division of Geriatrics and Centre of Excellence in Aging and Chronic Disease, McGill University, Montreal, Canada; 15 Department of Kinesiology, University of Western Ontario, London, Canada; 16 Department of Psychiatry, Division of Geriatric Psychiatry and Department of Medicine, Division of Clinical Pharmacology, University of Western Ontario, London, Canada; 17 Robarts Clinical Trials Inc, London, Canada; 18 Centre de Recherche, Institut Universitaire de Gériatrie de Montréal, Montréal, Canada; 19 Department of Sports Studies, Bishop’s University, Sherbrooke, Canada; 20 Faculty of Medicine, University of Montreal, Montréal, Canada; 21 St. Joseph’s Health Care, London, Canada; 22 Montreal Heart Institute, Research Centre, Montreal, Canada

**Keywords:** MCI, Exercise, Cognitive training, Vitamin D, Cognition, Gait, Dementia

## Abstract

**Background:**

Physical exercise, cognitive training, and vitamin D are low cost interventions that have the potential to enhance cognitive function and mobility in older adults, especially in pre-dementia states such as Mild Cognitive Impairment (MCI). Aerobic and progressive resistance exercises have benefits to cognitive performance, though evidence is somewhat inconsistent. We postulate that combined aerobic exercise (AE) and progressive resistance training (RT) (combined exercise) will have a better effect on cognition than a balance and toning control (BAT) intervention in older adults with MCI. We also expect that adding cognitive training and vitamin D supplementation to the combined exercise, as a multimodal intervention, will have synergistic efficacy.

**Methods:**

The SYNERGIC trial (SYNchronizing Exercises, Remedies in GaIt and Cognition) is a multi-site, double-blinded, five-arm, controlled trial that assesses the potential synergic effect of combined AE and RT on cognition and mobility, with and without cognitive training and vitamin D supplementation in older adults with MCI. Two-hundred participants with MCI aged 60 to 85 years old will be randomized to one of five arms, four of which include combined exercise plus combinations of dual-task cognitive training (real vs. sham) and vitamin D supplementation (3 × 10,000 IU/wk. vs. placebo) in a quasi-factorial design, and one arm which receives all control interventions. The primary outcome measure is the ADAS-Cog (13 and plus modalities) measured at baseline and at 6 months of follow-up. Secondary outcomes include neuroimaging, neuro-cognitive performance, gait and mobility performance, and serum biomarkers of inflammation (C reactive protein and interleukin 6), neuroplasticity (brain-derived neurotropic factor), endothelial markers (vascular endothelial growth factor 1), and vitamin D serum levels.

**Discussion:**

The SYNERGIC Trial will establish the efficacy and feasibility of a multimodal intervention to improve cognitive performance and mobility outcomes in MCI. These interventions may contribute to new approaches to stabilize and reverse cognitive-mobility decline in older individuals with MCI.

**Trial Registration:**

Identifier: NCT02808676. https://www.clinicaltrials.gov/ct2/show/NCT02808676.

**Electronic supplementary material:**

The online version of this article (10.1186/s12877-018-0782-7) contains supplementary material, which is available to authorized users.

## Background

Over 46 million people lived with dementia worldwide in 2015, with 1 new case every 4.1 s [1]. The cost associated with these dementia cases is over $800 billion US [1]. There is no cure for dementia. Recently, there has been an important shift in interventional studies on dementia to targeting early stages or pre-dementia states. Mild cognitive impairment (MCI) is thought to be an intermediate state between normal cognition of aging and very early dementia and, as a pre-dementia state, is commonly regarded as the optimal stage to intervene with preventive strategies and early treatments [2, 3]. Promising interventions for people with MCI include physical exercise, cognitive training, and vitamin D supplementation.

Physical exercise, specifically aerobic exercise (AE) and progressive resistance training (RT), have been demonstrated to improve cognitive outcomes, along with improved physical capacity and mobility in older adults [4, 5]. Both, AE [6] and RT [7] trials have reported positive results in improving cognitive performance, with consistent findings also observed after AE interventions lasting more than 3 months [4, 8]. RT has been studied less extensively than aerobic training in older adults, particularly in Mild Cognitive Impairment (MCI).

Similarly, cognitive training (e.g., computer based cognitive process training) may improve cognition, mobility, and postural control in older adults. Recent systematic reviews have shown cognitive benefits of computer-based cognitive training [9, 10]. Notably, a dual-task cognitive training regimen designed by our group has demonstrated that this type of training can also improve balance in healthy older adults [11]. Recent research suggests that improvements in brain plasticity occur after cognitive training [12, 13].

Vitamin D deficiency has been associated with cognitive dysfunction, dementia and mobility decline in older adults [4, 14–16]. Vitamin D is a neurosteroid hormone that exhibits neuroprotective attributes through antioxidative mechanisms, neuronal calcium regulation, immunomodulation, enhanced nerve conduction, and detoxification mechanisms [14, 17–20]. Compelling evidence from animal models and epidemiological studies supports a potential beneficial role for vitamin D on cognitive function [18, 21].

Robustly designed trials with longitudinal follow-up have been recommended to investigate the comparative benefits of isolated and multi-domain interventions in MCI to improve cognition and function [22]. To date, the effect of combined AE and RT in MCI is unknown. Moreover, the added value of adding cognitive training and vitamin D supplementation to physical exercise for improving global cognition, executive function, and memory in MCI has not been assessed. [22] The SYNERGIC TRIAL (SYNchronizing Exercises, Remedies in GaIt and Cognition) is designed to evaluate the effect of the combined exercise (AE and RT), alone or in combination with cognitive training and vitamin D supplementation, in older adults with MCI. This trial is being conducted by the Motor Exercise and Cognition Team (MEC Team 12) of the Canadian Consortium on Neurodegeneration in Aging (CCNA), part of the Canada Dementia Strategy.

### Hypotheses

1. Twenty weeks of supervised combined exercise (AE and RT) will significantly improve cognitive function in older adults with MCI, as assessed by primary outcome Alzheimer’s Disease Assessment Scale-Cognitive (ADAS-Cog 13 and plus modalities) and secondary outcomes, compared to a balance and toning (BAT) control.
2. Adding cognitive training to combined exercise will significantly improve primary and secondary outcomes compared to combined exercise without cognitive training.
3. Adding vitamin D supplementation to combined exercise will significantly improve primary and secondary outcomes compared to combined exercise without vitamin D supplementation.
4. The multi-domain intervention (combined exercise + cognitive training + vitamin D supplementation) will significantly improve primary and secondary outcomes compared to the control intervention.

## Methods/Design

### Design

The SYNERGIC TRIAL (SYNchronizing Exercises, Remedies in GaIt and Cognition) is a randomized, phase II, five-arm, double-blind controlled study evaluating the effect of combined exercise with and without cognitive training and vitamin D supplementation on cognitive function. A total of 200 participants with MCI, aged 60 and older will be enrolled and randomized into one of five arms:

Arm 1: combined AE and RT exercise + cognitive training + vitamin D.

Arm 2: combined AE and RT exercise + cognitive training + placebo D.

Arm 3: combined AE and RT exercise + control cognitive training + vitamin D.

Arm 4: combined AE and RT exercise + control cognitive training+ placebo D;

Arm 5: BAT exercise + control cognitive training + placebo D.

Note: The active interventions are in bold. Arm 5 includes only control interventions.

**Fig. 1:**
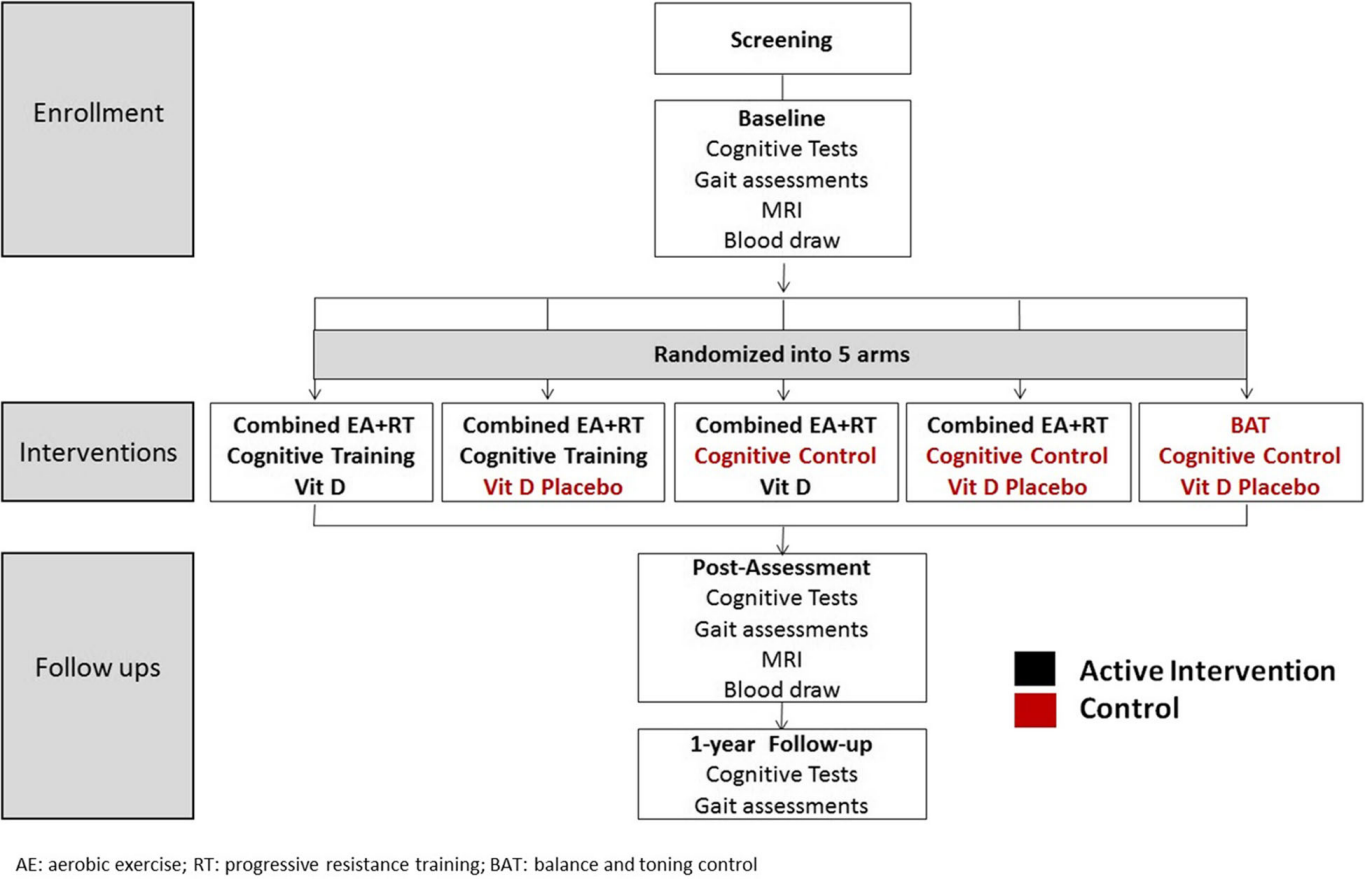
Consortium flowchart for the SYNERGIC Trial

**Fig. 2:**
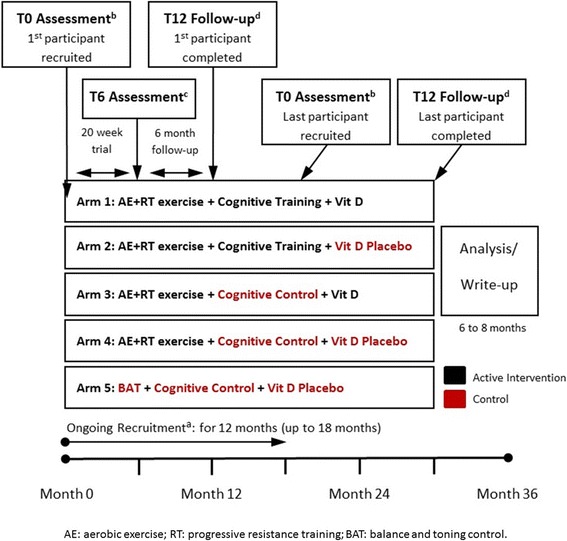
Schematic Timeline of the SYNERGIC Trial. **a** Recruitment of participants will be an on-going process with individuals being assigned to groups as they are enrolled. Recruitment is expected to be finished between 12 to 18 months. **b** Baseline assessments (T0) will be completed within one-week of participant starting the specific intervention/placebo. Participants will return to the clinic six months (**c**) after starting the specific intervention/placebo to complete the post intervention assessment (T6). After six months participants will return to the clinic again (T12) at which time their final assessment will be completed (**d**). 9 months after their first baseline assessment (T0) i.e. 3 months post-intervention, there will be a follow-up phone call

Figure 1 illustrates the trial design and Fig. 2 summarizes the timeline of the trial consisting of an approximate 12–18 months enrolment period, and 12 months of follow-up. The trial adheres to the Consolidated Standards of Reporting Trials guidelines for the conduct and reporting of clinical trials, as extended to non-pharmacologic interventions [23].

### Setting

Interventions will be done across five sites: London (lead and sponsor site), Waterloo-University of Waterloo, Waterloo-Wilfrid Laurier University, Montreal, and Vancouver. Participants will be recruited primarily from the community and from clinics serving MCI populations.

### Participants

#### Eligibility

The target recruitment is 200 older adults aged 60 to 85 years old with MCI who meet inclusion and exclusion criteria as outlined here (London target recruitment: 40 participants, Waterloo-University of Waterloo target recruitment: 20, Waterloo-Wilfred Laurier University target recruitment: 20, Montreal target recruitment: 50, and Vancouver-University of British Columbia target recruitment: 70). Although age, sex, and education will be included as covariates in the analytical models, recruitment will not be stratified.

#### Inclusion criteria

Participants must meet each of the following criteria for enrolment into the study:

1. Age 60 to 85 years old.
2. Self-reported levels of proficiency in English, or French (at Montreal site only), for speaking and understanding spoken language.
3. Able to comply with scheduled visits, treatment plan, and other trial procedures.
4. Able to ambulate at least 10 m independently.
5. Having MCI defined following Albert et al. [2] criteria:

a- Subjective cognitive complains,
b- Objective cognitive impairment in one of the following four cognitive domains: memory, executive function, attention, and language, operationalized using one or more of the following: MoCA Test (Montreal Cognitive Assessment) with scores ranging from 13 to 24/30, Logical Memory below Alzheimer’s Disease Neuroimaging Initiative (ADNI) cut-offs (< 9 for 16+ years of education; < 5 for 8–15 years of education; < 3 for 0–7 years of education), Consortium to Establish a Registry for Alzheimer’s Disease (CERAD) word list recall < 6.
c- Preserved activities of daily living operationalized as a Score > 14/23 on the Lawton-Brody Instrumental Activities Of Daily Living (IADL) [24] scale and confirmed by clinician’s interviews.
d- Absence of dementia using criteria from the Diagnostic and Statistical Manual of Mental Disorders, Fifth Edition [25] and/or Global Clinical Dementia Rating (CDR) ≤ 0.5 [26].
6. Having normal or corrected to normal vision in at least one eye so that they can identify symbols and stimuli presented on a computer screen in front of them.
7. Must be in sufficient health to participate in the study’s exercise training program as determined using the Physical Activity Readiness Questionnaire-Plus (PARQ+) screening tool, coupled with evaluation by a certified exercise physiologist and/or a physician for clearance to participate in combined exercise training program.

#### Exclusion criteria

Participants who exhibit any of the following conditions are to be excluded from the study:

1. Serious underlying disease which, in the opinion of the investigator, may preclude engagement in interventions or may interfere with the participant’s ability to participate fully in the study.
2. Participant with uncontrolled major depression, schizophrenia, severe anxiety or drug abuse.
3. Current parkinsonism or any neurological disorder with residual motor deficits (e.g. stroke with motor deficit), active musculoskeletal disorders (e.g. severe osteoarthritis of lower limbs) or history of knee/hip replacement affecting gait performance at clinical evaluation.
4. Intention to enroll in other clinical trials during the same time period.
5. Active participation in an exercise program involving AE or RT regimen 2 or more times per week in previous 6 months.
6. Taking vitamin D, cognitive enhancers, neuroleptics, or anticholinergics.
7. Known hypercalcemia and/or disorder that is cause of hypercalcemia (e.g. hyperparathyroidism/ Paget’s disease/ Sarcoidosis).
8. Known renal/kidney insufficiency.
9. Uncontrolled hypertension.
10. Uncontrolled diabetes.

#### Sample size

Sample size calculation is based on changes in our primary outcome: global cognitive function measured using the Alzheimer Disease Assessment Scale Cognitive 13 and the plus modality (ADAS-Cog 13 and plus). Changes in the ADAS-Cog test has been used as primary outcome in pharmacological clinical trials in dementia [27] and in 2 previous studies testing the effect of exercises on cognition in MCI participants [28, 29]. A total of 200 participants, with 160 in the combined exercise (pooled from the first 4 arms in one group) and 40 participant in the BAT control group, would have 80% power to detect an effect size (mean difference divided by the standard deviation) of 0.5 at 2-sided, 5% significance level. In addition, two-group comparisons with 80 participants per group would have 88% power to detect an effect size of 0.5 at the 5% significance level. The effect size of 0.5 was selected based on previous studies showing an effect size of exercise on ADAS-Cog ranging from 0.6 to 0.3 in MCI populations. [28–30]. The sample size estimations may be regarded as conservative, as the final data will be analyzed using analysis of covariance (ANCOVA) to adjust for baseline ADAS-Cog scores and other important patient characteristics.

### Measures

Test performed in the screening session, and during three visits (baseline, 6 months, and 12 months) are itemised in Table 1. Information concerning demographics, chronic diseases, comorbidities, chronic medications, history of previous falls, fear of falling, and balance confidence will be recorded using valid questionnaires at baseline. Additional descriptors to be tested include the activities of daily living using the Alzheimer Disease Cooperative Study Activities of Daily Living inventory (ADCS-ADL), Lawton-Brody IADL, the Short Form quality of life questionnaire (SF-36), the Generalized Anxiety Disorder 7 (GAD 7), Geriatric Depression Scale (GDS-30), CDR, the physical activity scale for the elderly (PASE), and the Mini Mental State Examination (MMSE).

**Table 1:** Assessments across study visits for SYNERGIC Trial

Procedure	Visit 1 Screening	Visit 2 Baseline	Visit 3 6 month	Telephone Follow-up at 9 month	Visit 4 12 month
Written Informed Consent	X				
Demographic Information	X				
Mini Mental State Examination (MMSE)	X				
Physical Activity Readiness Questionnaire Plus (PARQ +)	X				
Logical Memory 1 & 2	X				
CERAD Word List Recall	X				
PASE Questionnaire	X				
Montreal Cognitive Assessment (MoCA)	X		X		X
Generalized Anxiety Disorder 7 (GAD-7)	X		X		X
Geriatric Depression Scale (GDS-30)	X		X		X
Clinical Dementia Rating (CDR)	X		X		X
Activities of Daily Living (ADCS-ADL and IADL)	X		X		X
Clinical Medical Questionnaire		X	X		X
Dual Task Control Assessment		X	X		X
ADAS-Cog 13 (+ tests ^a^)		X	X		X
Trail Making Test A & B ^a^		X	X		X
Digit Symbol Test ^a^		X	X		X
Digit Span Forward and Backward WAIS-III ^a^		X	X		X
Boston Naming Test ^a^		X	X		X
Verbal Fluency Test ^a^		X	X		X
Colour Word Interference Test		X	X		X
Quality of Life Questionnaire (SF-36)		X	X		X
Short Physical Performance Battery (SPPB)		X	X		X
Gait Assessment using Gait Mat and accelerometers (when available)		X	X		X
Six Minute Walk Test (6MWT) ^b^		X	X		X
Neuroimaging (MRI)		X	X		
Blood Draw		X	X		
Falls Calendar ^c^		X	X	X	X

^a^ Testing included in the ADAS-Cog plus

^b^ This test may be completed at the gym facility on the first day of intervention

^c^ Calendar will be given to participant to complete and will be submitted to Research Staff at exercise training

#### Primary outcome

Changes in cognitive function will be assessed using the ADAS-Cog in two modalities, the 13 items modality (ADAS-Cog 13) and the plus modality (ADAS-Cog plus, Table 1) [25]. Improvement in either modality is considered evidence of efficacy. The ADAS-Cog 13 is a scale that consists of 13 brief cognitive tests assessing memory, language, attention, concentration and praxis. Scores range from 0 to 84, with higher scores indicating higher severity of cognitive impairment [25]. The ADAS-Cog has been used a primary outcome measure in numerous trials with MCI and Alzheimer’s Disease (AD) [28, 29]. The ADAS-Cog plus has marked advantages over the ADAS-Cog 13 as an outcome measure in MCI populations since it incorporates items concerning executive function [25]. The following tests are adding to the ADAS-Cog 13 to comprise the plus modality: Trail-Making Test (TMT) A & B, the WAIS-R Digit Symbol Substitution Test (DSST), the Digit Span forward & backward, and Category Fluency. In brief, the TMT (A & B) is a two part test, which assesses attention, speed, and mental flexibility and has been widely used clinically for assessing deficits in attention and executive functioning [31]. Trails A, where participants connect numbers in ascending order is truncated at 3 min and Trails B, where participants connect numbers and letters in ascending and alternating order is truncated at 5 min. Psychomotor speed is assessed with the DSST [32], which evaluates the speed with which participants copy arbitrary symbols associated with corresponding digits, by referring to a number-symbol key at the top of the page. The Digit Span test is an auditory attention task, where participants are asked to recall a series of numbers forward and backward. For category fluency, a measure of speed and flexibility of verbal thought, participants are asked to name as many items as possible in a specified category (vegetables & animals); unique responses during the first minute in each category are counted.

We expect that our interventions, over 20 weeks, will show improvement and/or less decline in cognition as measured by the ADAS-Cog (13 and plus modalities) at month 6 and at month 12**.** Significant changes in either time points will be considered preliminary evidence of efficacy. Additionally, reducing the proportion of participants, expressed as percentages per allocated group, with abnormal ADAS-Cog scores (dichotomous variable, cut-off score to be determined with ADAS-Cog literature) after intervention will be considered evidence of efficacy. Furthermore, we expect a significant statistical improvement in the transformed score of ADAS-Cog plus using an algorithm validated by Crane et al. [33].

#### Secondary outcomes

Secondary outcomes include neuropsychological assessments, gait and mobility outcomes (including incidence of fall), neuroimaging, and blood biomarkers.

#### Cognitive outcomes

Secondary cognitive outcomes will include MoCA test for global cognition, the recall list from the ADAS-Cog to evaluate verbal semantic memory, TMT A and B, DSST, Digit Span Test (Forward and Backwards), Boston Naming Test, Verbal fluency (animals and vegetables), and Colour Word Interference Test (Table 1). We expect improvement in participants of the active intervention in these cognitive tests, with the larger improvement in the multimodal intervention.

### Gait outcomes

#### Gait velocity and variability under single and dual-tasking

Gait velocity will be assessed as the time taken to walk 6 m using an electronic walkway system (ProtoKinetic® and/or GAITRite® Systems, Inc.). Gait variability of spatial and temporal gait variables (stride time, stride length, double support time and step width) will be calculated using the coefficient of variation (CV = (standard deviation / mean) × 100). The CV is a standardized measure of variability allowing comparison of gait variables measured in different units, having different means and range of values. Gait walks will be performed 3 times under single-task conditions, and one time under each of the three dual-task conditions (described below), and one time as fast gait. Dual-tasking assessments will permit calculation of dual-task cost for all gait variables of interest [34, 35]. Specifically, we expect participants in the combined exercise intervention to increase their gait velocity, decrease their gait variability and reduce their dual-task cost for the gait variables of interest.

Gait performance will be recorded using electronic walkway systems which automatically determine spatiotemporal gait parameters from imbedded sensors activated by foot pressure [36]. The gait mat will be located in a well-lit room with start and end points marked on the floor 1 m from either end of the mat. Participants will perform three main tasks: 1) preferred walking speed, 2) dual-task walking (counting backwards by 1’s, subtracting 7’s, and naming animals out loud while walking) and 3) fast walking. In all walks, participants will start 1 m before the beginning of the 6-m walkway and continue to travel 1 m past the end of the walkway. This procedure is in place to ensure steady-state walking and to minimize any effects of acceleration and de-acceleration during the course of the walk [37, 38]. The dual-task conditions selected are based on previous research which demonstrated that counting backwards requires both working memory and attention [39] and naming animals is related to verbal fluency, which relies on semantic memory [39, 40]. The evaluator will record any counting errors during walking so that it can be compared with the same mental tasks while seated. The seated assessments will be timed at 10 s and will be performed in the beginning of all cognitive assessments (before ADAS-Cog) to prevent practice effects in dual-task gait performance. Reliability has been previously established for this protocol in people with MCI [41] and an instructive video can be found at the “www.gaitandbrain.com/resources” as the *“Guidelines for Gait Assessments in CCNA”.*

#### Falls

A fall is defined as ‘unintentionally coming to rest on the ground, floor, or other lower level and not due to a seizure, syncope, or an acute stroke’ [42]. Events caused by overwhelming environmental hazards (e.g., being struck by a moving object) are not considered a fall. Recurrent falls are defined as ‘two or more events in a 12-month period’. Falls will be recorded throughout the 12-month trial, participants will be provided with a falls calendars, on which they will record any falls that have occurred, and they will be asked to bring them monthly to the training sessions to review with a research staff member. After the completion of the intervention, participants will be contacted at month 9 and 12 to report incidental falls. We expect participants in the combined intervention to reduce the number of falls compared to the sham intervention.

#### Mobility assessments

To further evaluate mobility, participants will be performing the Short Physical Performance Battery (SPPB), and the 6 min walk test (6MWT). We expect participants in the active intervention to present the larger improvements.

### Neuroimaging

Brain Magnetic Resonance Imaging (MRI) will be performed at baseline and 6-month visit. The imaging protocol will follow the *Canadian Dementia Imaging Protocol* developed for CCNA and available at http://www.cdip-pcid.ca. Pre-post regional patterns of brain plasticity will be assessed using structural (high-resolution 3D T1-weighted images = 7 min) in order to get voxel-based volumetric and cortical thickness measures, as well as diffusion tensor imaging (DTI, 30 directions, with AP/PA correction scan = 7 min) to calculate fractional anisotropy and diffusivity in white matter tracts as well as derive tract-based statistics. Cerebrovascular integrity and pathology will be assessed using the following contrasts: PD/T2 = 5 min; FLAIR = 7 min, and T2* = 5 min). Functional magnetic resonance imaging at rest (rsfMRI plus field map = 12 min) will also be acquired to measure change in data-driven functional networks. Scanning time will take 1.3 h. We expect that all active interventions will improve brain morphology (structural MRI), chemistry (spectroscopy MRI), and function (resting state MRI) compared with control interventions as defined by: increased hippocampal volume (mm^3^) by MRI scanning; positive localized Voxel-Based Morphometry (VBM) brain changes (z-score relative change); decreased total volume of White Matter Hyper-intensities (WMHs) (mm^3^); and lead to beneficial hippocampal and anterior and posterior cingulate MRS metabolite changes (% increase in N-acetylaspartate, and increase in phosphocreatine metabolites) and resting state MRI prefrontal activation.

### Biological markers

A blood draw will take place at baseline and 6-month visit in fasting conditions. Serum biomarkers of inflammation (C reactive protein, and interleukin 6), neuroplasticity (brain-derived neurotropic factor), endothelial health (vascular endothelial growth factor 1), and vitamin D serum levels will be measured before and after intervention. Samples will be collected, processed and stored securely at the respective site in a − 80 C freezer used for research purposes. We expect that active interventions will preferentially decrease inflammatory markers, increase brain-derived neurotrophic factor (BDNF) levels [29], and decrease vascular endothelial growth factor (VEGF-1) compared to either cognitive or control conditions.

### Study procedures

The screening visit (visit 1) will determine participant’s eligibility. There will be three assessment sessions: baseline, post-intervention at 6 months, and 12-month follow-up (Fig. 1). The baseline assessment will occur prior to randomization. Outcomes will be assessed by trained assessors blinded to group allocation. The measures at each assessment are described in Table 1 and timelines are shown in Fig. 2.

#### Randomization

Upon completion of the screening and baseline assessments, participants will be randomized and allocated to one of the 5 study arm. The randomization sequence of the participants will be generated centrally by the research pharmacist of the sponsor site using a central, web-based randomization service (www.randomizer.org) and will be specific for each site. The research pharmacy will assign the investigational product (vitamin D/matching placebo, as “kits”) in compliance with the randomization list(s) and allocate a sequential randomization number to each participant. A block randomization by 5 will be applied to ensure an appropriate balance of the characteristics of participants between each arm. Permuted blocks will be employed to ensure balance over time. After the baseline assessment, research personnel not involved in measurement or intervention will access the randomization list(s) to determine the arm allocation and institute the corresponding study interventions.

#### Blinding

In order to minimize a source of bias, this is a double-blinded study. Research personnel performing the outcome assessments at 6 and 12 month follow-ups will be blinded to group allocation. Participants will be blinded to the “active” intervention and study hypotheses. If it was medically necessary to un-blind a participant, the central research pharmacy will be contacted by the principal investigator to obtain the code.

### Study interventions

All participants will participate in three (3) group-training sessions per week for 20 weeks, under the supervision of trainers. Each session will last approximately 90 min and will be comprised of 30 min cognitive training or the cognitive training control followed by approximately 60 min of combined AE and RT or BAT control exercise. All participants will receive a monthly supply of either a vitamin D supplement (tablet 10,000 IU) or a placebo to be taken three times per week. All arms will have an equal volume and frequency of contact with trainers over the 12 months of the study. Interventions will be delivered in small groups of up to eight individuals. Each training group will have 1–2 trainers present each session to ensure a ratio of one trainer per four participants. To avoid potential imbalances in exposure time, control conditions for exercise and cognitive training will have the same duration as the active interventions.

#### Combined AE and RT exercise

The combined exercise intervention will be held in appropriate fitness facilities and take place between Monday and Friday, ensuring that it is not on three consecutive days. Staff trained and certified in exercise training will supervise all sessions. Difficulty of aerobic and resistance exercise will be tailored to their individual functioning level, with constant monitoring by the trainers.

Participants will start with a 10 min warm up, which includes: marching in place with arm swings, bum kicks, dynamic hamstring stretching, hula hoop circles, shoulder circles, arm reaches, torso twists, ankle circles, dynamic calf stretching, side stepping with wrist circles, split-step knee bends, and quarter squats. The RT portion will occur next and includes 5 exercises: leg press & leg flexion (lower body), chest press, lat pull, and seated row (upper body). These exercises are performed alternating between lower and upper body exercises, with the number of repetitions, sets, and rest being modified every 4 weeks to increase muscular strength. RT will progress from loads and volumes appropriate for endurance (first 8 weeks) to those appropriate for maximal strength gains (last 12 weeks). Training volume, intensity, and rest are standardized and described in Additional file 1: Table S1. Participants should reach exhaustion at the last prescribed repetition of the last set. Training prescription for all exercises follows American College of Sport Medicine guidelines for strength development in older adults.

Following the RT portion of each session, participants will complete 2 sets of 10 min of aerobic exercise. The mode of exercise can vary and may include walking, a variety of ergometers (treadmill, elliptical machines, cycling ergometers, rowing machines, etc.), and other forms of free aerobic exercises, as long as the cognitive load is minimized. Intensity will be monitored using a Borg scale (0–10) with a target of 5–6 for weeks 1–8, 6–7 for weeks 9–16, and 7–8 for the last 4 weeks (Additional file 2: Table S2).

Each session will end with a ten-minute cool down, which will consist of the following stretches (each held for 20–30 s); quadriceps stretch, hamstring stretch, calf stretch, 2 hip stretches, static torso rotation, seated side bend, back and shoulder stretch, chest stretch, triceps stretch, neck stretch.

#### BAT control exercises

Participants assigned to the BAT control exercise condition will take part in balance and toning exercises in groups of up to eight participants, supervised by a trainer [43–48]. The exercises will be devoted to improving muscle tone and flexibility, without improving strength, and cardiorespiratory capacity. Resistant load and number of repetitions will not progress across exercise sessions, unless participants were unable to complete required repetitions at the beginning of the intervention. The session will start with the same 10-min warm-up completed in the combined AE and RT group. This will be followed by 40 min of a variety of balance and toning exercises that will target the entire body. The sessions will include functional training (wall squats, standing calf raises, standing leg abduction/adduction, standing ball walk up & down, seated back row with resistance tube, shoulder retractions, wall push-ups, toe walking, heel walking, quarter squats, gluteus kickback holds, standing ball twists, shoulder circuits, yoga ball chest press, yoga ball shoulder abduction, yoga ball leg adduction squeeze), balance training (standing leg circumduction, tandem stance, single leg stance, tandem stance ball relay pass, partner ball pass in tandem stance, tandem forward & backward walking, toe taps on bench), agility training (4 step zig zag in place, 4 step zig zag walking, line and cone drills), and core training (core contractions seated on exercise ball, exercise ball seated ball pass, seated exercise ball marching, modified & full bug on floor). Exercises will be cycled in and out every 3 weeks to maintain variety and decrease likelihood of progression. The session will end with the same 10 min cool down stretches as for the exercise group.

#### Cognitive training

The cognitive training intervention will involve tablet based (iPad®, held on a stand on a table) dual-task training that requires participants to maintain and prepare for many response alternatives (working memory) and to share attention between two concurrent tasks (divided attention). Difficulty of cognitive training is tailored to their individual functioning level. The training uses a custom-written program developed for neuro-rehabilitation and has been used in previous research trials for cognitive [39, 40] and mobility outcomes [12].

Cognitive training will take place in groups of up to eight participants before the exercise training session. Cognitive training will be 30 min, at maximum, and each participant will complete the cognitive training at an individual desk space, in a quiet room, wearing headphones. Each session, participants will perform a two different visuo-motor tasks, each with their respective sets of visual stimuli (e.g., letters, numbers, animals, vehicles, fruits, celestial bodies) and respective hand-button correspondences (i.e., buttons that are to be tapped with the thumbs on either the right or the left side of the tablet). Participants are instructed to perform as fast as possible, while maintaining accuracy. Tasks will be performed both separately and concurrently so that task-set cost and dual-task cost can be isolated. At each session, task combination for the sets of stimuli will change (from a total 18 combinations). Training will also include online feedback as well as histogram of daily performances to encourage improvement.

#### Control cognitive training

The cognitive training control group sessions will last a maximum of 30 min to align with the same time frame as the cognitive training group. Participants will alternate between 2 different tasks (touristic searching using internet and video watching) completed using the same tablet (iPad®) as in the cognitive training group. Sessions will be held in groups of up to eight participants, and in the first session they will receive a short introductory lesson on the use of the tablet and how to navigate the internet. For the touristic searching using internet, participants will be required to find 3 hotels, 3 touristic places, and 3 restaurants of their own preference in a city assigned by the instructor (a new city will be selected each session). They will also need to include the respective addresses of those places on their log sheet. For the video watching task, participants will watch a National Geographic video on YouTube selected by the instructor with a different video selected each session. They will watch the video for 20 min and during the remaining 10 min they will answer the following questions on their log sheet; 1) what is the video about; 2) what is the most important information in your opinion; 3) create a question based on the video and answer your own question. Despite completion of the tasks, participants will be stopped at 30 min.

#### Vitamin D supplementation

Participants will receive vitamin D supplementation (1 tablet of 10,000 IU of vitamin D_3_) three times per week in order to reach a weekly cumulative dose of 30,000 IU per week (equivalent of 4258 IU daily). The vitamin D capsules will be provided by the research coordinator in vials at the first session and every 4 weeks for a total of five vials during the training period. Vials will be returned by the participants to the research coordinator at the end of each 4 week block. *Rationale and bio-safety of the dose:* The dose of 10,000 IU/day is currently approved by Health Canada as a supplementation for older adults. Heaney et al. have administered doses of 30,000 IU/day vitamin D_3_ to adult men for 5 months, with no significant changes in serum calcium concentrations or adverse reactions [49–51]. A comprehensive review of toxic effects of vitamin D found that the lowest level at which an adverse effect was observed was a serum calcidiol concentration of 200 nmol/l, corresponding to a daily intake of 40,000 IU. Therefore, weekly doses of 30,000 IU have a 9-fold weekly margin of safety of the established safe dose.

#### Placebo vitamin D supplementation

Participants allocated to placebo vitamin D will receive a placebo capsule that will perfectly match the vitamin D tablets. They will take one tablet three times per week, to match what the active vitamin D participants will be taking. The vials will be dispensed on a 4 week basis in the same manner as the vitamin D tablets.

### Data analysis

#### Planned analysis

Descriptive statistics for demographic and baseline characteristics will be provided with means and standard deviations, or medians and the interquartile range where appropriate, for continuous characteristics, and frequencies and percentages for categorical variables. Analysis will be conducted as intention-to-treat (ITT) and as per-protocol analysis (PPA). Observation of a statistically significant difference in the primary outcome at post-intervention time point will be considered preliminary evidence of efficacy. Contrasts from linear mixed models containing the variable for intervention arm, the categorical variable for time points, and their interaction term will be used for comparing continuous outcomes such as ADAS-Cog scores, adjusting for baseline outcome values and patient characteristics including age, sex, and education. This approach can readily handle three time points (baseline, 6 months and 12 months) and thus may be regarded as a generalization of the ANCOVA. Similar analyses for binary outcomes such as reduction of prevalence rate of abnormal ADAS-Cog plus score will be performed with generalized linear mixed effect models. Specific contrasts from the mixed-effects models will be constructed to test the following hypotheses. The first hypothesis (exercise intervention) will be evaluated by collapsing arms 1–4 (exercise intervention, *n* = 160) and comparing them with arm 5 (no exercise intervention, *n* = 40). For the second hypothesis (cognitive intervention), arms 1–2 (cognitive intervention, *n* = 80) and arms 3–4 (no cognitive intervention, *n* = 80) will be collapsed and compared. For the third hypothesis (vitamin D intervention), we will collapse arms 1 and 3 (vitamin D intervention, *n* = 80) and compare them with collapsed arms 2 and 4 (no vitamin D, *n* = 80). For the fourth hypothesis (synergic effect), arm 1 (all active interventions, *n* = 40) will be compared with arm 5 (all control interventions, *n* = 40).

For the secondary outcomes, 6 month changes in gait performance and secondary cognitive outcomes of interest will be performed using one way analyses of variance. For group comparisons that are statistically significant, pairwise comparisons will be made using Tukey’s multiple comparisons tests. Secondary analyses will be also performed according to an intention-to-treat principle and per-protocol analysis. An economic analysis will also be performed.

All statistical tests will be two-tailed, and a *p*-value of less than 0.05 will be considered to indicate statistical significance. All calculations will be made using SPSS (SPSS version 23.0, SPSS Inc., Chicago, IL) and Stata (Stata Statistical Software: Release 14. College Station, TX: StataCorp LP.).

#### Frequency of the analyses

Preliminary analysis will be performed after finishing recruitment to ascertain descriptive characteristics at baseline assessment. Interim efficacy analyses will be performed when recruitment is reaching 50% of target sample and final efficacy analysis will be performed at the end of the trial since no safety issues are anticipated in this study.

#### Adverse event and serious adverse event reporting

Adverse events will be recorded for subjects starting at the time of signing the Informed Consent until their discontinuation of the study. All adverse events will be recorded on an ongoing paper log, which will be reviewed by each site leader and forwarded to the team leader at London site, who will determine both the intensity of the event and the relationship of the event to study procedures, and monitored until resolved. Based on the outcome it will be up to the team leader to medically determine if it is justified for the participant to continue in the study or terminate their participation. A serious adverse event is defined as any incident that is unexpected, and related or possibly related to participation in the research study.

#### Rescue medication and risk management

Higher doses of vitamin D may lead to hypervitaminosis D manifested by hypercalcemia and its sequelae. Treatment of acute or chronic intoxication includes withdrawal of vitamin D3 and any calcium supplements, maintenance of a low calcium diet and if needed corticosteroids or calciuric diuretics, such as furosemide and ethacrymic acid to decrease serum calcium concentrations. All participants will be monitored by site physicians and trained staff, and should any adverse events arise, participants will have access to a phone number and e-mail address provided on the copy of their signed consent form. They will be informed to use these contacts to notify the Principal Investigator about concerns that may be associated with treatment received in each site.

### Ethical considerations

This study is conducted in compliance with International Conference on Harmonization Good Clinical Practice (ICH-GCP) and all applicable regulatory requirements. Sponsor site obtained approval of the Research Ethics Board at the University of Western Ontario (REB# 107670), the Lawson Health Research Institute’s Clinical Research Impact Committee (R-15-038), and Health Canada (HC file - HC6–24-c195918 / HC protocol #201619) prior to initiating study-related activities. Each intervention site has also obtained local ethical approval.

## Discussion

Older adults with MCI are at a high risk of progression to dementia syndromes with incident rates often ten times higher than the cognitive healthy counterparts. Additionally, older adults with MCI have an increased risk of falling and mobility decline [52, 53]. Physical exercise, cognitive training, and vitamin D supplementation are emerging and promising non-pharmacological interventions to enhance mobility and cognitive functioning in older adults, especially in pre-dementia states such as MCI.

These three interventions have been tested separately, with positive results for physical exercise and cognitive training in improving cognitive function, and controversial evidence for vitamin D supplementation [8, 19]. Importantly, it is currently unknown whether combining AE with RT exercise has a better impact on cognitive performance than a BAT exercise modality, and if an integrated approach including combined AE and RT exercise in addition to cognitive training and vitamin D will pose a synergistic efficacy for improving cognition and mobility when compared with combined exercise alone.

**Fig. 3:**
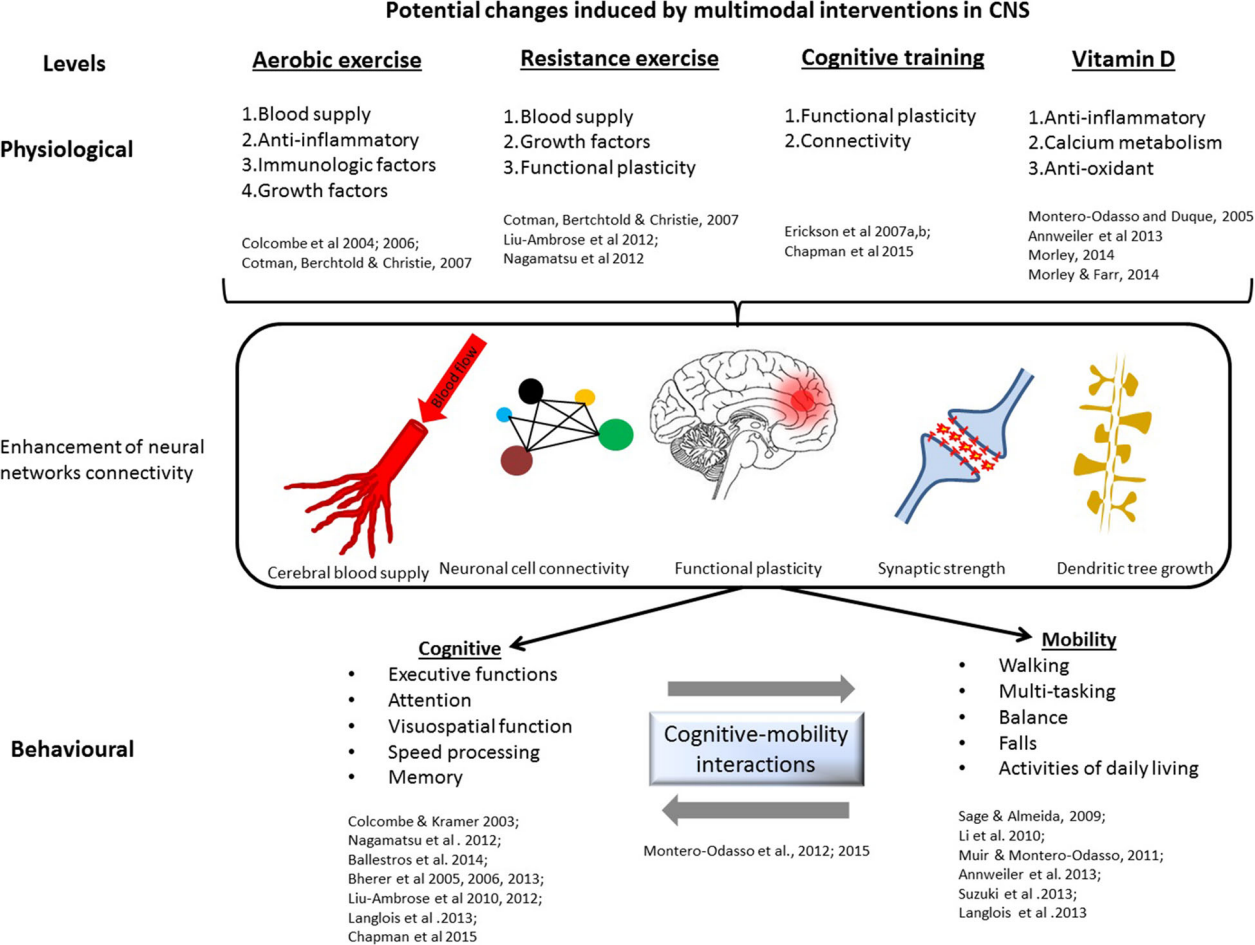
Conceptual model for individual and synergistic effects of planned interventions in the SYNERGIC Trial

Mechanistically, AE and RT exercises can provoke a cascade of biochemical, physiological, and structural changes in the brain, as summarised in Fig. 3. For example, AE increases blood flow, neurotrophic factor release, neurogenesis, immune system efficacy and metabolism. These effects of exercise could combat inflammatory processes and the atrophy of brain structures both often associated with aging and dementias [6, 22]. Interventions using RT exercises have found substantial improvements in high-order cognition (e.g. executive functions), whereas low-order cognition (e.g. attention, processing speed) is less benefited [6]. The reason for this selective improvement in cognition is unknown, but it is hypothesized that areas in the brain that modulate executive functions are more susceptible to both aging and physical exercises interventions. Mechanism suggested involve modulation of insulin-like growth factor-1 and insulin sensitivity, decreasing inflammation, enhancing release of brain-derived neurotrophic factor pathways, and even decrease brain amyloid load [7, 54, 55]. Combined exercise interventions have also shown increased brain volume and muscle mass in older adults [44].

Cognitive training can also improve cognition through enhancing brain functioning. Individuals who practiced monitoring of two tasks at the same time (i.e. dual-task training) on computer devices presented improved connectivity between prefrontal and temporal cortices, areas known to be important for executive functioning and memory, when compared to control participants [13]. Furthermore, imaging in these participants showed increased activity in these cortical areas during resting state, as shown by increased blood flow. With this, implementing a dual-task cognitive training program in older adults has the potential to selectively improve high-order cognitive functioning through brain plasticity and improved activation.

Epidemiological evidence suggests that serum levels of 25-hydroxyvitamin D below 50 nmol is associated with impaired executive functions and the development of dementia [18]. Similarly, vitamin D levels in serum have been associated with worse performance in the cognitive motor interface in MCI populations. The reason of this association remains to be determined; however, vitamin D (25-hydroxyvitamin D) supplementation has been hypothesized to cause enhancement of neuroprotective agents that decrease biochemical processes in the brain that accelerate cell death [19]. Activation of vitamin D receptor inhibits the production of *amyloid-β* (Aβ) protein in the brain. Deregulated production of Aβ protein influences a chain of biochemical mechanisms that increases the rate of hippocampal cell death and synaptic loss. Calcium function can also be regulated by vitamin D via down regulation of *L-type voltage-sensitive calcium* channels decreasing apoptosis. Finally, vitamin D can stabilize mitochondria leading to a reduction in oxidative damage. Thus, we hypothesized that vitamin D could modulate cognitive functioning through three major pathways: Aβ inhibition, calcium metabolism and mitochondrial activity [56]. Additionally, we have also hypothesized that as a consequence of cognitive enhancement with vitamin D supplementation, motor function may also be improved [16, 57].

This randomized controlled trial is the first of its kind to test whether a multimodal intervention combining AE and RT with or without cognitive training or vitamin D supplementation can improve cognition and mobility related outcomes in older adults with MCI. Strengths of our protocol include the selection of a comprehensive battery of assessments sensitive to mobility-cognitive changes, as determined in a previous Pan-Canadian Consensus in Gait and Cognition (available at www.gaitandbrain.com/resources), and a population target, older adults with MCI, which is thought to be the ideal stage to intervene to delay cognitive decline before dementia.

Results for this study will provide data concerning the effect size of the proposed multimodal interventions compared with isolated interventions in cognitive and mobility outcomes in MCI. We expect that the combined exercise intervention will provide the larger effect size contributing in the changes in cognitive outcomes followed by the effects of cognitive training and vitamin D supplementation.

In conclusion, the SYNERGIC Trail may contribute to establish the efficacy of an integrated therapeutic strategy, a multimodal approach, to stabilize and reverse cognitive decline in older individuals with MCI, and help to delay progression to dementia syndromes. The proposed interventions are aimed at improving the quality of life of many older adults with significant cognitive decline and also alleviate economic burdens on health care. It is estimated that 500,000 older Canadians have MCI [1] and even a modest one-year delay in dementia incidence could save the Canadian Health System over $109 billion over 30 years [58].

## Additional files

Additional file 1: **Table S1.** Participants in the SYNERGIC Trial will complete the following resistance training three times per week for 20-weeks. (DOCX 14 kb)

Additional file 2: **Table S2.** Participants in the SYNERGIC Trial will complete the following aerobic training three times per week for 20-weeks. (DOCX 14 kb)

Additional file 3: **Table S3.** Members and affiliations of the *Canadian Gait and Cognition Network*. (DOCX 16 kb)

## Abbreviations

6MWT: 6 Minute Walk Test
AD: Alzheimer’s disease
ADAS-Cog: Alzheimer’s disease assessment scale-cognitive
ADCS-ADL: Alzheimer disease cooperative study activities of daily living
ADNI: Alzheimer’s disease neuroimaging initiative
AE: Aerobic exercise
ANCOVA: Analysis of covariance
Aβ: amyloid-β
BAT: Balance and toning
BDNF: Brain-derived neurotrophic factor
CCNA: Canadian consortium in neurodegeneration and aging
CDR: Clinical dementia rating
CERAD: Consortium to establish a registry for alzheimer’s disease
CV: Coefficient of variation
DSST: Digit symbol substitution test
GAD 7: Generalized anxiety disorder 7
GDS-30: Geriatric depression scale
IADL: Instrumental activities of daily living
ICH-GCP: International conference on harmonization good clinical practice
ITT: Intention-to-treat
MCI: Mild cognitive impairment
MMSE: Mini mental state examination
MoCA: Montreal cognitive assessment
MRI: Magnetic resonance imaging
PARQ+: Physical activity readiness questionnaire-plus
PASE: Physical activity scale for the elderly
PPA: Per-protocol analysis
rsfMRI: Functional magnetic resonance imaging at rest
RT: Resistance training
SF-36: Short form quality of life questionnaire
SPPB: Short physical performance battery
SYNERGIC: SYNchronizing Exercises, and Remedies in GaIt and Cognition
TMT: Trail-making test
VBM: Voxel-based morphometry
VEGF: Vascular endothelial growth factor
WMHs: White matter hyper-intensities
