# Supplementary material for: SYNERGIC TRIAL (SYNchronizing Exercises, Remedies in Gait and Cognition) a multi-Centre randomized controlled double blind trial to improve gait and cognition in mild cognitive impairment
Source: BMC Geriatr. 2018 Apr 16;18:93. doi: 10.1186/s12877-018-0782-7 (PMC5902955; doi:10.1186/s12877-018-0782-7)
Supplement: Supplementary file 1 — Table S1. Participants in the SYNERGIC Trial will complete the following resistance training three times per week for 20-weeks. (DOCX 14 kb) [file 12877_2018_782_MOESM1_ESM.docx]

**Table S1** Participants in the SYNERGIC Trial will complete the following resistance training three times per week for 20-weeks.

| Weeks | Sets | | | Repetitions | | | Rest between sets (sec) |
| --- | --- | --- | --- | --- | --- | --- | --- |
|  | Day 1 | Day 2 | Day 3 | Day 1 | Day 2 | Day 3 |  |
| 1-4 | 2 | 2 | 2 | 15-18 | 15-18 | 15-18 | 30 |
| 5-8 | 3 | 2 | 3 | 12-15 | 12-15 | 12-15 | 30 |
| 9-12 | 3 | 2 | 3 | 10 | 10 | 10 | 60 |
| 13-16 | 2 | 3 | 2 | 8 | 12 | 8 | 60 |
| 17-20 | 3 | 2 | 3 | 6 | 8 | 6 | 60 |

Sets indicate the number of times the participant should repeat the duration of the exercise.
