# Supplementary material for: SYNERGIC TRIAL (SYNchronizing Exercises, Remedies in Gait and Cognition) a multi-Centre randomized controlled double blind trial to improve gait and cognition in mild cognitive impairment
Source: BMC Geriatr. 2018 Apr 16;18:93. doi: 10.1186/s12877-018-0782-7 (PMC5902955; doi:10.1186/s12877-018-0782-7)
Supplement: Supplementary file 2 — Table S2. Participants in the SYNERGIC Trial will complete the following aerobic training three times per week for 20-weeks. (DOCX 14 kb) [file 12877_2018_782_MOESM2_ESM.docx]

**Table S2** Participants in the SYNERGIC Trial will complete the following aerobic training three times per week for 20-weeks.

| Weeks | Sets | Duration (min) | Intensity (Borg 0-10) | Rest between sets  (min) |
| --- | --- | --- | --- | --- |
| 1-4 | 2 | 10 | 5-6 | 1 |
| 5-8 | 2 | 10 | 5-6 | 1 |
| 9-12 | 2 | 10 | 6-7 | 1 |
| 13-16 | 2 | 10 | 6-7 | 1 |
| 17-20 | 2 | 10 | 7-8 | 1 |

Sets indicate the number of times the participant should repeat the duration of the exercise.
