# Supplementary material for: SYNERGIC TRIAL (SYNchronizing Exercises, Remedies in Gait and Cognition) a multi-Centre randomized controlled double blind trial to improve gait and cognition in mild cognitive impairment
Source: BMC Geriatr. 2018 Apr 16;18:93. doi: 10.1186/s12877-018-0782-7 (PMC5902955; doi:10.1186/s12877-018-0782-7)
Supplement: Supplementary file 3 — Table S3. Members and affiliations of the Canadian Gait and Cognition Network. (DOCX 16 kb) [file 12877_2018_782_MOESM3_ESM.docx]

**Table S2** Members and affiliations of the *Canadian Gait and Cognition Network*.

| **Canadian Gait and Cognition Network** | | | |
| --- | --- | --- | --- |
| **Province** | **City** | **Institution** | **Member** |
|  |  | Gait and Brain Lab, Schulich School of Medicine & Dentistry. University of Western Ontario | Manuel Montero-Odasso |
|  |  | Schulich School of Medicine & Dentistry. University of Western Ontario | Robert Bartha |
|  |  | Schulich School of Medicine & Dentistry. University of Western Ontario | Michael Borrie |
|  |  | Regional Mental Health Care-London. University of Western Ontario | Amer Burhan |
|  |  | Schulich School of Medicine & Dentistry. University of Western Ontario | Vladimir Hachinski |
|  |  | School of Physical Therapy. University of Western Ontario | Susan Muir-Hunter |
| Ontario | London | School of Kinesiology. University of Western Ontario | Kevin Shoemaker |
|  |  | Epidemiology and Biostatistics. University of Western Ontario | Mark Speechley |
|  |  | Schulich School of Medicine & Dentistry. University of Western Ontario | Luciano Sposato |
|  |  | Pharmacy department. St Joseph’s Health Care | Leanne Vanderhaeghe |
|  |  | Department of Psychiatry. University of Western Ontario | Akshya Vasudev |
|  | Ottawa | Faculty of Health Sciences, Interdisciplinary Scholl of Health Sciences. University of Ottawa | Sarah Fraser |
|  |  | The Sun Life Financial Movement Disorders Research and Rehabilitation Centre. Wilfrid Laurier University | Quincy Almeida |
|  | Waterloo | Faculty of Applied Health Sciences, Department of Kinesiology. University of Waterloo | William McIlroy |
|  |  | Faculty of Applied Health Sciences, Department of Kinesiology. University of Waterloo | Laura Middleton |
|  |  | Department of Psychology; Centre de recherche, IUGM. Concordia University | Louis Bherer |
|  |  | Division of Geriatric Medicine. McGill University / Université McGill | Olivier Beauchet |
|  | Montréal | Unité de Neuroimagerie Fonctionnelle Centre de recherche, IUMGo. Université de Montréal | Julien Doyon |
| Québec |  | Department of Psychology. Concordia University | Karen Li |
|  |  | Division of Geriatric Medicine. McGill University / Université McGill | José A. Morais |
|  | Quebec City | Department of Rehabilitation , Universite Laval | Bradford J. McFadyen |
| Alberta | Edmonton | Glenrose Rehabilitation Hospital. University of Alberta | Richard Camicioli |
| British Columbia | Vancouver | Aging, Mobility, and Cognitive Neuroscience Lab, Department of Physical Therapy. University of British Columbia | Teresa Liu-Ambrose |
| New Brunswick | Fredericton | Faculty of Kinesiology and Institute of Biomedical Engineering. University of New Brunswick | Christopher McGibbon |
| Pennsylvania | Pittsburgh | Department of Biomedical Informatics. University of Pittsburgh | Ervin Sejdic |
